# Women’s experiences of the consenting process for pregnancy remains disposal following early miscarriage

**DOI:** 10.1136/bmjsrh-2023-201982

**Published:** 2024-01-05

**Authors:** Susie Kilshaw

**Affiliations:** Department of Anthropology, University College London, London, UK

**Keywords:** abortion, spontaneous, qualitative research, reproductive health

## Abstract

**Background and methodology:**

UK clinical practices around managing pregnancy remains after pregnancy loss involve a process of documenting consent. Women are typically offered options for disposal, which may include cremation, burial, releasing for private arrangements, releasing to a funeral director and, in some cases, sensitive incineration. A single researcher conducted 20 months of ethnographic fieldwork in one National Health Service (NHS) Trust including observing the consenting process for pregnancy remains disposal (n=28) and interviewing 27 women, including 19 who had experience of the consent process for pregnancy remains disposal, about their understanding, attitudes and experiences of pregnancy remains disposal. Transcripts were analysed for representative themes.

**Results:**

Prior to the discussion and consenting process most participants had not given consideration to disposal methods. Participants expressed surprise about the discussion and disposal pathways with most suggesting it was inappropriate, particularly given the early stage of their pregnancy (<12 weeks’ gestation). In some cases, the consenting process caused distress due to the way the participant framed their pregnancy remains being divergent from implied meaning in discussions about disposal.

**Conclusions:**

Current practices appear discordant with the views of some women experiencing miscarriage. A person-centred approach to pregnancy remains disposal is recommended to accommodate a diverse range of approaches so as not to challenge a woman’s experience of and agency about her body, pregnancy and pregnancy remains.

**WHAT IS ALREADY KNOWN ON THIS TOPIC**

- Pregnancy remains disposal is a sensitive issue, but one that has become a key element of miscarriage care pathways. Research suggests that current procedures are not in keeping with women’s needs.

**WHAT THIS STUDY ADDS**

- This research provides in depth understanding of women’s experience of the consenting process for pregnancy remains disposal in one National Health Service (NHS) Trust.

**HOW THIS STUDY MIGHT AFFECT RESEARCH, PRACTICE OR POLICY**

- The research can contribute to revisions of local NHS policy and practice. The research findings can inform revisions of guidance at a national level to provide more consistency in the information sharing and documenting for pregnancy remains disposal.

## Introduction

In the UK, the Human Tissue Authority (HTA) guidance on the disposal of pregnancy remains^1^ and those of other relevant bodies such as the Royal College of Nursing (RCN)^2^ and National Bereavement Care Pathway (NBCP)^3^ inform local National Health Service (NHS) practice. Such governance outlines that women should be given options (burial, cremation, and sensitive incineration for the disposal of their pregnancy remains.^1–3^ [NB. Sensitive incineration involves the packaging, storage, and incineration of pregnancy remains separately from other clinical waste. The HTA and RCN regard sensitive incineration as an appropriate method and recommended it being offered to women as an option, yet many clinical settings do not provide this option, including in all of Scotland where the local government deemed it unacceptable in any circumstance.] Although consent for disposal is not required, due to pregnancy remains being legally considered the woman’s tissue,^1 2^ most NHS Trusts use consent forms to ensure an audit trail.

There is a lack of research in the UK and globally on women’s understanding about the disposal of tissue following the end of a pre-viability pregnancy. Several qualitative studies have produced insight into women’s experience of miscarriage and other pregnancy endings; however, few have addressed the concerns women may have about the management of tissue. [NB. Pregnancy endings is a more inclusive term to include miscarriage, stillbirth, terminations, ectopic pregnancy and molar pregnancy. It also encapsulates the complexity and nuance of women’s experiences.] Women’s opinions regarding donation of fetal tissue for research purposes^4 5^ has been explored, finding women wanted reassurance that the fetus no longer existed in any material form.^4^ Research into women’s experiences following an elective abortion^6^ found women wanted access to information but did not favour being required to make decisions about disposal. Previous research found consent to be a key issue for both professional and parent participants in the emotionally charged area of perinatal pathology.^7^ The ‘Death Before Birth’ (DBB) project^8–10^ assessed the impact of the HTA guidance on practices around disposal and found good and improving practice, but significant variation with women’s needs not always being met. While the DBB team interviewed women who had experienced a range of pregnancy endings and disposal processes, this was often some time after the experience. The current research aimed to focus on women who had very recently experienced miscarriage in one NHS Trust to gain indepth understanding about their experience of pregnancy remains disposal and the consenting process to assess whether current practice was meeting women’s needs.

There are contradictions in legal categories, institutional practices, and national guidance related to pregnancy remains and the fetal body,^5 10 11^ with research showing these practices may not resonate with lived experience and subjective classifications. If a woman miscarries outside of clinical settings, they may treat the pregnancy remains as they wish with most flushing them, as revealed by the research. Trust policies are guided by the view, first expressed in Polkinghorne, that based on its potential to develop into a human being, a fetus is entitled to respect, broadly comparable to that of a living person.^8^ Such an approach implies the fetus has value in and of itself. Hospital practices such as ceremonial disposal frame miscarriage as the death of a baby regardless of what women think of the material,^10^ meaning women who do not want fetal personhood recognised are obliged to take part in practices which enact it.^11^ The author was unable to identify any research that focused on the consenting process for the disposal of miscarried pregnancy remains. This anthropological research sought to explore women’s understandings, attitudes and experiences of disposal of their pregnancy remains. In particular, the aim was to understand patient’s experience of consenting by interviewing and observing women who had undergone at least one miscarriage.

## Methods

### Study design and participants

The ethnographic research included extensive fieldwork including observations, interactions and semi-structured interviews with patients experiencing miscarriage and those involved in their care over a 20-month period (table 1). Women were recruited at one NHS Trust in England. Semi-structured interviews explored interactions with pregnancy remains, the meaning it holds, and experiences of disposal in both clinical and domestic settings. This article reports on one aspect of the study design: 28 observations of the consenting process for pregnancy remains disposal and interviews with 19 of the participants who had experience of the consenting process (ie, 17 participants had experienced the consenting process and two were shown the consent form with permission during the follow-up interview to gain their perception of the form). There is a variation in the UK with different clinical settings having different approaches to remains disposal. The approach of the host clinic was fairly typical. Participation was offered on posters and flyers displayed in the site and by clinic staff familiar with the study who handed the study information sheet to women. Women eligible for inclusion were: (1) 18 years of age or older, (2) diagnosed with a miscarriage in the previous 6 months, (3) able to provide written consent, (4) willing to comply with the study protocol and (5) English-speaking. Women were excluded if they: (1) were unable to provide written consent, (2) cognitively impaired to a degree or in a way which would mean that they were not able to tell their story or (3) were experiencing recurrent miscarriage (defined as more than three consecutive miscarriages). The research was granted full ethics approval.

**Table 1:** Ethnographic research project study design (comprising 20 months of fieldwork)

Research method	Value (n)
**Observations**	
Patient assessments, diagnosis, treatment and follow-up appointments	60
Discussion and consent procedure for remains disposal	28
**Semi-structured interviews**	
Women experiencing miscarriage	37
During 2020–2022 fieldwork	27
Encountered consenting process via:	19
Surgical management of miscarriage	13
Miscarried in clinic (manual removal of pregnancy tissue)	2
Miscarried at home, remains taken to clinic	2
Miscarried at home, flushed remains, shown documents by researcher	2
During 2014–2016 fieldwork (previous participant cohort)*	10
Healthcare professionals	38
Host NHS Trust staff	24
Clinicians (doctors, nurses, midwives)	14
Other clinical staff	4
Other staff (counsellors, chaplaincy team, bereavement team)	6
NHS staff from other clinical settings	7
Other professionals (ie, funerary and crematorium)	7

It is difficult to convey extensive ethnographic research in quantitative tabular form, but Table 1 details some of the fieldwork activities. The research included informal interactions and discussions with NHS staff involved in miscarriage care and observations inside and outside the clinic settings, including remains disposal pathways.

*Participants from the principal investigator's previous project who had been treated in the same NHS Trust in 2014/2015 were revisited to explore the long-term residues and remains of miscarriage to explore their experience of pregnancy remains disposal.

NHS, National Health Service.

Women self-referred, and after expressing interest via email or telephone call, potential participants were again sent the study information sheet and consent form and given an opportunity to ask questions. Informed consent to participate was sought and recorded prior to interview. Interviews were conducted by a trained medical anthropologist (SK) from April 2020 to September 2023; the research was suspended from June 2021 to May 2022 due to the COVID-19 pandemic. Women were interviewed during or soon after their miscarriages and follow-up interviews were conducted 3–6 months later. While the research included miscarriages up to 22 gestational weeks, all but one participant experienced first-trimester miscarriage, and this is the focus of the article.

Most interviews were conducted in person, but due to COVID restrictions a small number were conducted online. Interviews lasted 45–90 minutes. Individual, semi-structured interviews were conducted as ‘guided conversations’^12^ and respondents were encouraged to give their own accounts and meanings in relation to the main research questions: their experience of the remains and remnants of miscarriage broadly defined and their experience associated with disposal of pregnancy remains. Fieldnotes were recorded after each interview.

Audio recordings were professionally transcribed verbatim. Transcripts were reviewed for accuracy and familiarity through repeated and careful reading. Fieldnotes and interview transcripts were analysed using a thematic analysis approach; the method was iterative and based on a grounded theory approach.^13^ Participants were assigned an enrolment number and pseudonym.

Observations were not recorded and no personal information was collected. Staff were informed of the research and invited to participate. Participating staff would provide women with the study information and seek verbal consent for observation. If both agreed the researcher would be invited into the session. Some participants took part in both observations and interviews.

### Patient and public involvement

The overall research project was informed by the author's previous work on miscarriage, particularly interviews with patients experiencing miscarriage and their discussions about their pregnancy remains, and her ongoing work with a miscarriage patient tutor group.

## Results

Between April 2020 and September 2022, 27 women made contact agreeing to participate and were subsequently interviewed (table 2). No participants withdrew consent. Participants volunteered a variety of reproductive histories, and many had experienced earlier pregnancy endings including miscarriages, ectopic pregnancies, abortions and one stillbirth. These experiences were often used by participants as points of resonance or comparisons, and several participants had had experiences of both home miscarriage and clinically managed miscarriage. The analysis is organised under four themes (see table 3 for additional supporting quotations).

**Table 2:** Demographic characteristics of the study participants

Characteristic	Value
Age, years (mean (range))	35.4 (25–47)
Ethnicity (n (%))	
White	22 (81)
Asian	3 (11)
Black, Black British or Black African	2 (7)
Disposal option chosen (n)	
Home	
Flushed/put in bin	7
Home burial*	2
Private cremation	1
Took into clinic	2
Clinical setting	
Hospital disposal (burial)	13
Private arrangement	
Cremation	1
Home burial	1
First pregnancy (n)	12
Previous pregnancies (n)	15
One previous miscarriage (n)	1
Two or more previous losses (including stillbirth, ectopic, miscarriage) (n)	9
Previous pregnancies ended in live birth (n)	5

*One woman flushed most of the pregnancy tissue, but saved the final “clot” and buried it.

**Table 3:** Examples of participant quotations supporting each theme

Theme	Example excerpt
Positive experience of care	Everyone at [the EPAU]) was great. I was handled with such care. [Annabel] I’ve been to the EPU and [the hospital] for various things… the majority of the people I have come across they are so hard working and under so much pressure…and they come to you and it is like all of that other crap hasn’t happened. They are just focused on you and they aren’t afraid to touch you even during corona times and they sit with you. Although it has been really crap I have been able to see goodness in people… definitely felt cared for and understood. [Marianne] [The hospital] and [nurse] just did what she said she was going to do. She was very personal and very friendly. Nothing was too much for her. Even though she’s clearly very, very busy and she kind of went above and beyond. … I went in on the Friday and the staff were very friendly and didn’t make me feel silly for being upset and they were just very considerate. [Rose] They were lovely, and you are not supposed to have anyone in there but I was obviously distraught, so they sort of let Matt come in with me, which was really kind of them and spoke to [nurse] and she was just lovely obviously, just sort of explaining the situation, giving me the options, letting me cry, a lot, and then sort of letting me go off and figure out what to do really. [Scarlett] I feel like I was given very good care and I was very well taken care of. [Shirley] Everyone is terribly kind… the people at [the EPAU] were beautifully kind. [Mary] I think the [EPAU] is amazing… with my first two miscarriages it wasn’t there. So I used to have to go and wait with all the pregnant women which is just so wrong. [Melanie]
Unprepared	They signpost you … they say “Here's all the information about natural management, expectant management, surgical management”, they've given you all of that information, so they're telling you all about that and what to expect so why not just put [information about disposal] with all that information. At least if people have got time to just digest it…. I'd much prefer that than on the day just be like “Here's this form, you’ve got to decide whether you want to put it in a mass grave” or whatever it said, the horrific stuff it said on there. I just couldn’t believe it was on there. [Nell] And certainly for me, how I, you know, how I cope with things is by being really prepared. And I cope with things by having all the information, knowing all the things and preparing myself for what’s going to happen and probably over reading about stuff and over researching it…. So I guess when it came to that consent form, I wasn’t prepared and I didn’t know they were going to ask that. And I suppose as well one of the ways I had been coping with the whole miscarriage, it was very early, I was maybe 6 or 7 weeks. For me, at that point, with my personal belief system and my scientific personal beliefs, is it’s not a baby. It’s a clump of cells that has a lot of hopes and dreams attached to it and it could be a baby, but it’s not a baby. [Scarlett] So, I wasn’t expecting to be handed that form. So, I was like, “What? You’re asking me what you want” – I just assumed you’d have the surgery and I don’t know, it’s just dealt with. [Nell] Do you know what I’d not even thought about it. I was shocked because for me it feels very medical. It feels like you’re going in to get an operation, it’s just run of the mill. Even though it’s not because I was very upset and I was very shocked. I was upset. But yes, it just seems very strange, it feels like chalk and cheese… I just felt like, yes surgery, get it over and done with. What do you want to do with your remains of your baby? I don't know. It caught me off guard and actually I felt really bad… Yes, it was weird and I think it was weird because I never had the option or I never felt like I had the option the first time as well, so it was a shock. [Laura]
Time (proximity to surgery) (miscarriage journey (gestational stage/fetal development)	For me that wasn’t the right point to bring it up, when I was about to swallow some pills and put myself, essentially on to a kind of operating table. You have to sort of psyche yourself up for these procedures, particularly if you know you’re going to have something done under local and there’s potential for it to be painful… the focus of my attention was preparing myself for that. So then to have the consent in there to talk about the remains just, it really threw me. [Meredith] It was overwhelming… you know you're going to have this operation. And it takes a long time to have it done. And then you’ve got to wait… and I know how busy they are and there's lots of people there and I think that probably makes it a bit worse. So, by the time you go in there you’ve keyed yourself up: Is the operation going to uncomfortable? What are they going to do? Am I going to hear it all? Is it going to be horrendous? And then there's a long list of things they have to go through about the risks to me and stuff like that. And it’s like this is tacked on the end but you’ve just been given so much information it’s overwhelming. And then they suddenly say, “Oh, and by the way what do you want to do with the baby?” What, what? So, maybe having that information in advance might have been you know, “Would you like to do this”, and then they could say, “Did you have a chance to read the information?” That might have been less nervy. [Lisa] Do you know what, I’d not even thought about it. I was shocked because for me it feels very medical. It feels like you’re going in to get an operation, it’s just run of the mill. Even though it’s not because I was very upset and I was very shocked… But yes, it just seems very strange, it feels like chalk and cheese. It feels like two completely different things. I guess, yes, there's not that many occurrences where surgery and then something so sensitive is paired together. I just felt like, yes surgery, get it over and done with. What do you want to do with your remains of your baby? I don't know. It caught me off guard. [Laura] I sort of wondered afterwards… whether there’s an option to kind of decouple that, you know. Because actually if I could have signed off that, you know I would have signed it off long ago… It/s also just something that you’ve maybe moved on from… they take you back to it… seeing those reminders of the actual, that moment of loss. That’s what drags you back… for me it was so far down the line that it was, it was just… yeah it wasn’t the right moment. [Meredith] I think because it’s already been 4 or 5 weeks. I think it might have been 5 weeks from the point where I had a scan to when I had the surgery. So, I think, by the time I had the surgery, I was so – I already felt like I had processed it. And I know people deal with it completely differently, but I think I just felt like I was over it. Just wanted to move on and wanted it to be done with. [Nell] They came out and said about going into… not a mass grave, they didn’t say it like that but the communal grave…. for the weeks that I was pregnant… [option of taking home or releasing to funeral director] seemed an odd option…. And I just remember thinking that just sounds horrific but you don't have an option, I don't want to bring it home because I don't want to do that. And I don't think a funeral director is needed, if it had been … a fully formed fetus then yes, I would have done it. [Lisa] The [miscarried pregnancy] was just a bunch of cells… Whereas now I'm pregnant and I can feel her moving and I know that she looks like a baby and at the 12-week scan she looked a baby. I was like, Hmm I wonder how I'd feel about that consent form if I was shown that at 24 weeks? If I was shown that at 24 weeks I'm sure I would feel completely different about it… and I remember us talking about that there should be two different forms. I feel really strongly about that now, because I think the way that I felt then… if I was to put myself in that position where I was 20 weeks, 21 weeks or whatever, they need to be completely different because what's inside you is completely different. So, I feel quite strongly about that… If its someone that's going through it at 7 weeks, 8 weeks, and someone that's going through that at 20 weeks, they're two completely different experiences and should be dealt with separately. [Nell] Maybe it needs to be up to a certain point in gestation I don't know, but maybe there's a cut-off – the right to have it treated as medical waste. Because I’m very uncomfortable with the way that it was handled. [Scarlett] For me it was too early for anything… it wasn’t far enough ahead in my mind to be a baby… I know people that have lost babies at 20 weeks, for me that is more physically a baby then, it looks like a baby… whereas, as the stage I’ve been losing them it doesn’t really look like anything. [Rose] I suppose I felt that there wasn’t, it’s not that something wasn’t alive but it certainly wasn’t a baby to me at that point…. I think because we both felt… it wasn’t a baby, it wasn’t a child. We didn’t feel that there was a need for a more formal ritual around a goodbye sort of I don't know, a burial or cremation. … So, yes I suppose there was a surprise about the first options I suppose just because for me it wasn’t like a baby. [Nina]
Conflicting framings	And so, I wasn’t expecting that form. I hadn’t even thought about it in that way. By that point, I wasn’t still thinking about it being a baby or fetus. You know, it was 8 weeks. It had stopped developing. So, it’s hardly anything anyway. And I think to help me process it was how you think about it. It was just tissues. There’s nothing. So, I was a bit taken aback when I saw the form and I was like, oh my God, you’re talking about mass graves and people take it home. I don’t know. It freaks me out. It freaked me out a bit because I just wasn’t expecting to be asked what I wanted to do with it. I don’t know. And I guess it’s different, you know, if you’re much further along. I imagine it would feel completely different. Well, I know it would feel completely different. But I think because it was so early, I was just like - oh, I don’t know. I just felt really weird about it because it was so early. [Nell] One of the ways I had been coping with the whole miscarriage, it was very early, I was maybe 6 or 7 weeks. For me… with my personal belief system… is it’s not a baby. It’s a clump of cells that has a lot of hopes and dreams attached to it and it could be a baby, but it’s not a baby… So then being confronted with … that consent form and the treating of it as more than a small clump of cells, was just, it made it a lot more upsetting because it makes it a baby, which isn’t how I had been thinking of it…. So being asked to treat it like it was a baby suddenly became very upsetting. [Scarlett] [If I had miscarried at home] it would have gone in the bin like my sanitary towel. Like all the other ones, I just, I treated it like a period sort of thing. [Melanie] One of the doctors said it might be a blighted ovum… I found that really helpful because I felt like it didn’t even have the potential to be a baby because it had never done what it should do. ….[explained consent form would have been “traumatising” and said consent form made it] more meaningful, more tangible, more babylike and also therefore, much more traumatic. [Ruth]

### Positive experience of care

Patients were overwhelming positive about the care they received, saying they felt “understood”, “cared for”:

The staff there [Early Pregnancy Assessment Unit] provide such a brilliant service, not just in terms of the physical care but the emotional and psychological sides to everything as well.

It became clear in the initial coding phase of the research that consent for disposal was a key issue for both professional and patient participants and was commonly described as not in keeping with otherwise sensitive, responsive and excellent care.

Of the 27 women interviewed, 19 had experiences of the consent form most often in relation to the surgical management of their miscarriage (table 1) either to remove the entire pregnancy or “retained products of conception” when a miscarriage was incomplete. While four women welcomed the discussion and disposal options, 15 women felt the approach was inappropriate and, at times, unwelcome. Five women reported being distressed by the process, with more cases observed during fieldwork.

### Unprepared

Women consistently described being unprepared for the discussion about pregnancy remains disposal. Except in one case, despite encountering healthcare professionals previously, the issue was not broached until the day of the procedure:

[H]ow I cope with things is by being really prepared. … So I guess when it came to that consent form, I wasn’t prepared and I didn’t know they were going to ask that… And I think that is why I found it so upsetting. [Scarlett]

And to be honest that completely surprised me. I did not realise there was going to be discussion, I thought… they take it out and it goes down a tube into medical waste… it almost bothered me that I had to make that decision because I had never thought about it… I would have preferred not to have the choice. [Alex]

The participant quoted above and others indicated they would have preferred to opt out of the discussion but found this was not possible.

Of the four participants who had described a positive experience, two had engaged in information-gathering and decision-making about their pregnancy material prior to their procedure by looking online and speaking to friends. These two women raised the issue of disposal prior to the consenting process.

### Inappropriate timing, chronology and ontology

The inappropriateness of the timing of the procedure was described in three ways: timing on the day of treatment, timing in relation to miscarriage care pathway, and in reference to gestational time. Introduced to a discussion about disposal when they were anxious and overwhelmed with information in relation to surgery was deemed inopportune, as was timing in relation to the chronology of their miscarriage journey. Women described the point of diagnosis as critical in the experience of loss. However, the consenting process coincided with surgical management of the miscarriage, which often occurred weeks after diagnosis due to women often opting for conservative or medical management first:

In my head I was done with it and then I started having to think about it. And the emotion is high again. [Alex]

Discussions about disposal were an upsetting reminder of the moment of loss from which the participant had begun to recover. One participant had reframed her miscarriage and its ongoing health implications as an illness:

Then to have the consent… to talk about the remains… it really threw me… because I think for a while now it hasn’t been a pregnancy for me, it’s been an illness, a chronic illness that I’ve been dealing with and trying to get better from. [Meredith]

The discussion was deemed inappropriate in relation the gestational stage and/or fetal development. In the case of early pregnancy, some women place their loss low on a hierarchical scale, note the absence of certain stages of developing personhood, such as hearing a heartbeat or witnessing movement during a scan.^14^ Women noted appropriateness of the disposal discussion had their pregnancies been further along and fetuses more developed:

[T]here wasn’t a real emotional attachment, that’s not to say I wasn’t upset, there was a lot of crying and upset about what could have been but not really upset about what it *was* at that point…. For me it wasn’t a baby loss. That’s very different. I think if it had been something like 22 weeks or something much later then it would have been a completely different response to it… while it’s early it’s nothing. …It’s a medical thing at this point. [Alex]

Here attention is drawn to the separation between sadness of loss and the biological material of miscarriage. For many women, particularly those experiencing pre-12-week miscarriage, the experience of loss is disentangled from the pregnancy remains. The material is not the fulcrum of grief and yet the practices around hospital disposal imply entanglement and, indeed, reify a link between the two.

#### Conflicting framings

At times consenting for disposal caused distress due to divergence of the meaning of the pregnancy remains with some associating it with biological matter or medical waste, expecting it to be treated as such:

Treat it like any other kind of thing that you’re removing from your body… maybe the part of me trying to process it was me being like, this wasn’t a baby…. It’s literally just some cells and tissues. So, then to be told, “We’re going to bury it”. Treating it like it is a human being is like totally opposite to how I’m trying to process it. [Nell]

Thinking of the material as waste or tissue was sometimes a means to cope with the experience and/or related to worldview. The consenting process challenged this protective framing:

One of the ways I had been coping… it was very early, I was maybe 6 or 7 weeks. For me… with my personal belief system… is it’s not a baby. It’s a clump of cells that has a lot of hopes and dreams attached to it and it could be a baby, but it’s not a baby… So then being confronted with … that consent form …. being asked to treat it like it was a baby suddenly became very upsetting… Like, you know, it’s not a baby and I shouldn’t be forced to confront it like that. [Scarlett]

The participant’s upset was due to the divergence in perceptions of her pregnancy material, with her view that hospital procedures were framing it as a baby, while she did not. Frictions derive from conflicting notions of the significance of pregnancy material and personhood.

## Discussion

These findings complement previous research which describes heterogeneous attitudes towards the end of a pregnancy, pregnancy material, and its disposal.^15 16^ This study provides detailed qualitative evidence of women’s experience of consenting for pregnancy remains disposal and shows it as an aspect of care that was consistently experienced as being problematic. In line with previous research, this study reveals that more could be done to ensure such processes meet women’s needs. A person-centred approach to understanding reproductive loss^15–18^ and remains disposal to accommodate diversity of meaning that individuals have about their pregnancy material is called for.

The HTA,^1^ RCN,^2^ NCBP^3^ and ICCM^19^ emphasise the importance of women being afforded the possibility not to have to make a decision about disposal. However, there is variation in how NHS settings manage this, with many not providing this possibility. Research found that in the few settings that this is offered, it is only a possibility for women undergoing a termination of pregnancy.^15^ The research shows that women have varied positions towards their pregnancy material, with some women experiencing miscarriage not seeing it as the loss of a baby and not wishing to have the material afforded value.

## Conclusions

As consent is not a legal requirement for pregnancy remains disposal, it is suggested that a revised approach be developed that de-couples the discussion and documenting of wishes from connotations of legality and traditional notions of informed consent. Information-sharing and documenting should occur earlier in the care pathway to prepare patients for discussions about disposal, and consideration be given as to whether disposal be integrated into the miscarriage care pathway.^8^ A tiered system could allow for women learning as much or as little about disposal options depending on their approach. Disclosure of disposal options should not be a ‘tick-box process’ but a discussion between the healthcare professional and the patient, which includes identifying what information the patient wants so as not to negatively impact their experience.^8^ The approach should allow flexibility and able to incorporate all approaches to pregnancy ends and pregnancy materials. It is recommended that revisions to current procedure include an opt-out so women are not to be forced to re-frame their miscarriage and pregnancy material in ways that are unwelcome or upsetting to them. A person-centred approach to pregnancy remains disposal would accommodate a diverse range of approaches and limit challenges to a woman’s experience of and agency about their body, their pregnancy and their pregnancy material.

## Data Availability

No data are available.

## References

[1] Human Tissue Authority (HTA) . Guidance on the disposal of pregnancy remains following pregnancy loss or termination. 2015. Available: https://www.hta.gov.uk/guidanceprofessionals/guidance-sector/post-mortem/guidance-sensitive-handlingpregnancy-remains

[2] Royal College of Nursing (RCN) . 2018. Managing the disposal of pregnancy remains: RCN guidance for nursing and Midwifery practice Available: https://www.rcn.org.uk/Professional-Development/publications/managing-the-disposal-of-pregnancy-remains-uk-pub-009-942

[3] National Bereavement Care Pathway . Miscarriage, ectopic pregnancy and molar pregnancy: full guidance document. 2022. Available: https://nbcpathway.org.uk/pathways/miscarriage-bereavement-care-pathway

[4] Pfeffer N . What British women say matters to them about donating an aborted fetus to stem cell research: a focus group study. Soc Sci Med 2008;66:2544–54. 10.1016/j.socscimed.2008.01.050 18375029

[5] Pfeffer N , Kent J . Consent to the use of aborted fetuses in stem cell research and therapies. Clin Ethics 2006;1:216–8. 10.1258/147775006779151210

[6] Myers AJ , Lohr PA , Pfeffer N . Disposal of fetal tissue following elective abortion: what women think. J Fam Plann Reprod Health Care 2015;41:84–9. 10.1136/jfprhc-2013-100849 25201907

[7] Reed K , Ferazzoli MT , Whitby E . "Why didn't we do it”? Reproductive loss and the problem of post-mortem consent. Soc Sci Med 2021;276:113835. 10.1016/j.socscimed.2021.113835 33780832

[8] McGuinness S , Kuberska K . Report to the Human Tissue Authority on disposal of pregnancy remains (less than 24 weeks’ gestational stage); 2017. Available: https://www.researchgate.net/publication/323552235_Report_to_the_Human_Tissue_Authority_on_disposal_of_pregnancy_remains_less_than_24_weeks’_gestational_stage

[9] Fuller D , McGuinness S , Littlemore J , et al . Preliminary project findings for meeting with representatives of the Department of Health and Social Care. 2018. Available: https://www.academia.edu/38633723/Preliminary_project_findings_for_meeti ng_with_representatives_of_the_Department_of_Health_and_Social_Care

[10] Kuberska K . Unwitnessed ceremonies: funerals for pre-24-week pregnancy losses in England. In: Kilshaw S , Borg K , eds. Navigating miscarriage: social, medical, and conceptual perspectives. Oxford, New York: Berghahn, 2020: 206–32. 10.2307/j.ctv1k3nqd4

[11] Middlemiss AL . Pregnancy remains, infant remains, or the corpse of a child? The incoherent governance of the dead foetal body in England. Mortality 2021;26:299–315. 10.1080/13576275.2020.1787365

[12] Lofland J , Lofland L . Analyzing social settings: a guide to qualitative observation and analysis. Belmont, CA: Wadsworth Publishing Company, 1984.

[13] Strauss A , Corbin JM . Basics of qualitative research: grounded theory procedures and techniques. Sage Publications, 1990.

[14] Middlemiss AL , Kilshaw S . Further hierarchies of loss: tracking relationality in pregnancy loss experiences. Omega (Westport) 2023:302228231182273. 10.1177/00302228231182273 37282837 PMC12444023

[15] Austin L , McGuinness S . Reproductive loss and disposal of pregnancy remains. NILQ 2019;70:131–53. 10.53386/nilq.v70i1.236

[16] Middlemiss AL . Invisible labours: the reproductive politics of second trimester pregnancy loss in England. New York, NY: Berghahn, 2024.

[17] Layne LL . Motherhood lost: a feminist account of pregnancy loss in America. New York, London: Routledge, 2003.

[18] McNiven A . (Re)Collections: engaging feminist geography with embodied and relational experiences of pregnancy losses [PhD dissertation]. Durham University, 2014

[19] Institute of Cemetery and Crematorium Management (ICCM) . The sensitive disposal of fetal remains: policy and guidance for burial and cremation authorities and companies. 2015. Available: https://www.iccm-uk.com/iccm/wp-content/library/iccm_Fetal%20Remains%20Policy%20Updated%20Sept2015%20.pdf

